# Nephroprotective Properties of the Glucose-Dependent Insulinotropic Polypeptide (GIP) and Glucagon-like Peptide-1 (GLP-1) Receptor Agonists

**DOI:** 10.3390/biomedicines10102586

**Published:** 2022-10-15

**Authors:** Tomislav Bulum

**Affiliations:** 1 Vuk Vrhovac Clinic for Diabetes, Endocrinology and Metabolic Diseases, University Hospital Merkur, Dugi dol 4a, 10000 Zagreb, Croatia; tomislav.bulum@kb-merkur.hr; 2 Medical School, University of Zagreb, Šalata 2, 10000 Zagreb, Croatia

**Keywords:** chronic kidney disease, diabetes, incretins, dual GLP-1/GIP receptor agonist

## Abstract

Diabetes mellitus is the leading cause of chronic kidney disease, and about 30–40% of patients with diabetes will develop kidney disease. Incretin hormones have received attention during the past three decades not only as a pharmacotherapy for the treatment of type 2 diabetes, but also for their cardiorenometabolic effects. The main incretins are glucagon-like peptide-1 (GLP-1) and glucose-dependent insulinotropic polypeptide (GIP). Additional to the pancreas, receptors for GLP-1 are widely distributed in various organs, causing positive effects on endothelial function and vascular atherogenesis. Along with glycemic control and weight reduction, GLP-1 receptor agonists also strongly improve cardiovascular and renal outcomes in patients with type 2 diabetes. Recently, a dual GIP and GLP-1 receptor agonist has been approved for the treatment of type 2 diabetes. Compared to GLP-1 receptor agonist semaglutide, dual GIP and GLP-1 receptor agonist tirzepatide showed a superior reduction in hemoglobin A1c and body weight. Preliminary results also suggest that tirzepatide improves kidney outcomes in adults with type 2 diabetes with increased cardiovascular risk. In this review, we present the nephroprotective properties of dual GIP and GLP-1 receptor agonists as a new drug to treat type 2 diabetes.

## 1. Introduction

**Figure 1:**
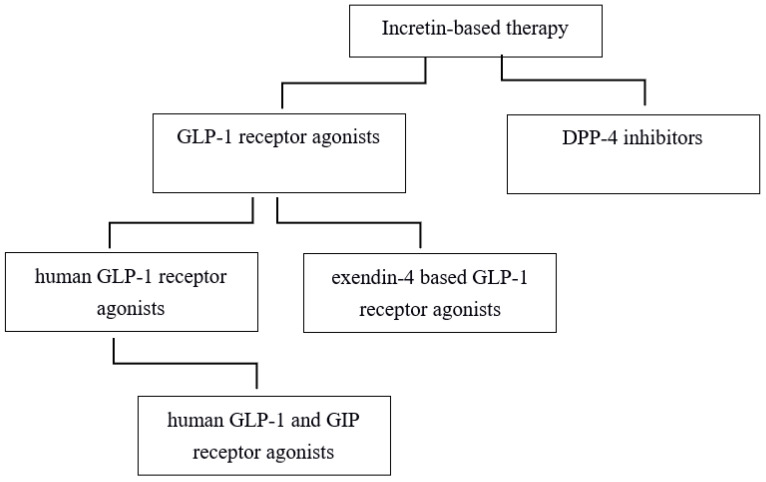
Incretin-based therapy.

Incretins are peptide hormones responsible for the so-called ‘incretin effect’ after oral glucose and food intake. After oral glucose intake, incretins are secreted from enteroendocrine K and L cells found in the intestinal epithelium [1]. This incretin effect phenomenon is responsible for the majority of insulin secretion from the pancreas, and also for the control of postprandial hyperglycemia after oral glucose and food intake compared to intravenous infusion of glucose avoiding the digestive tract [2]. The main incretins are glucagon-like peptide-1 (GLP-1) and glucose-dependent insulinotropic polypeptide (GIP) [3]. In patients with type 2 diabetes (T2DM), the incretin effect is diminished or completely lost [4]. Since GLP-1 infusion may restore the incretin effect in patients with T2DM, the development of two incretin drug classes has been achieved—the GLP-1 receptor agonists and the dipeptidyl peptidase 4 (DPP-4) inhibitors (Figure 1). Initially, GIP administration was not able to restore the incretin effect in patients with T2DM [5], and consequently, GIP has not been considered as an antidiabetic drug. Incretin-based therapy has been based on GLP-1 as a therapeutic agent for the treatment of T2DM. Additional to the effects on blood glucose, therapy with GLP-1 receptor agonists resulted in beneficial effects on metabolic conditions such as overweight and obesity, beneficial effects on renal and liver function, reduced cardiovascular risk, and also had a positive impact on the treatment of neurodegenerative disorders [6]. In recent years, combined therapy with GLP-1 and GIP has been successfully used in preclinical studies and later confirmed in human studies. It has been shown that simultaneous infusion of GLP-1 and GIP, compared with single GLP-1 or GIP infusion, additionally improves glucose control and weight loss [7]. Recently, a dual GIP and GLP-1 receptor agonist has been put on the market for the treatment of patients with T2DM [8]. Compared to GLP-1 receptor agonist semaglutide, dual GIP and GLP-1 receptor agonist tirzepatide has been reported to show a superior reduction in hemoglobin A1c and body weight [9].

Diabetes mellitus with hypertension is the major cause of chronic kidney disease, and the majority of patients with diabetes will develop kidney disease [10]. Diabetic nephropathy is the major cause of end-stage renal disease in western countries and the United States [11]. Hyperglycemia along with other metabolic disorders associated with metabolic syndrome and diabetes, causes specific initial kidney damage, such as glomerular sclerosis and hypertrophy, fibrosis, and tubulointerstitial inflammation [12]. Renal function is further damaged over time, resulting in glomerular hyperfiltration, progressive proteinuria and albuminuria, and a further decrease in glomerular filtration rate up to end-stage renal disease and dialysis [12]. In addition to the pancreas, receptors for GLP-1 are widely distributed in various organs, causing positive effects on endothelial function and vascular atherogenesis. GLP-1 receptors are present in kidneys, and along with their effects on glucose control and weight loss, have direct effects on natriuresis, renal inflammation, and oxidative stress [13]. GLP-1 receptor agonists are also used as a cardio and nephroprotective therapy. Compared to GLP-1 receptor agonist semaglutide, dual GIP and GLP-1 receptor agonist tirzepatide was found to show a superior reduction in hemoglobin A1c and body weight [9]. Preliminary results also suggest that tirzepatide strongly improves kidney function compared to insulin in adults with T2DM with increased cardiovascular risk. In this review, we present the nephroprotective properties of the dual GIP and GLP-1 receptor agonists as a new therapeutic option for patients with T2DM [14].

### 1.1. Glucagon-like Peptide 1

GLP-1 is a peptide produced by L cells in the terminal ileum and colon, and is secreted after food intake [15]. GLP-1 acts through its receptor, which is expressed in various tissues, including the pancreas, nervous system, kidneys, stomach and intestine, heart, lungs, muscle, adipose tissue, and skin, indicating an important function of GLP-1 in improving not only plasma glucose, but also the homeostasis of the whole organism [16]. By activating pancreatic receptors, GLP-1 stimulates insulin secretion depending on glucose concentration, and also inhibits the apoptosis of beta cells [17]. Moreover, upon activating pancreatic receptors, GLP-1 also inhibits the release of glucagon from alpha cells and inhibits glucose secretion from the liver [16]. GLP-1 effects on glucose control in patients with T2DM are equally influenced by beta cell insulin secretion and the inhibition of glucagon secretion from alpha cells [18]. Activation of GLP-1 receptors contributes to weight loss via vagal afferent stimulation, resulting in slower intestinal motility and gastric emptying, and also acts via the central nervous system, promoting satiety and reducing food intake [19,20]. Slower gastric emptying induced by GLP-1 also delays glucose influx into the circulation, and has important effects on postprandial hyperglycemia [21]. GLP-1 effects on fasting blood glucose are primarily mediated via effects on pancreatic islets [22]. GLP-1 upgrades cardiac output and vasodilatation in skeletal muscle and adipose tissue, resulting in increased glucose uptake in the muscles [23]. However, because of its short half-life, only small amounts (about 10–15%) of active GLP-1 are present in the circulation; this effects targeted organs because GLP-1, as well as GIP, is rapidly degraded within several minutes after secretion by the enzyme DPP-4, and because of their fast renal elimination [24].

To prolong the short half-life and its effects on peripheral GLP-1 receptors, two GLP-1 forms were designed as therapeutic agents in the treatment of patients with T2DM. The first GLP-1 receptor agonists that came on the market were based on exendin-4. Exendin-4 has a similar structure as native GLP-1, and was isolated from the saliva of the lizard *Heloderma suspectum*. Exendin-4 is resistant to degradation by the DPP-4 enzyme, therefore, the half-life of GLP-1 is significantly extended. Accordingly, the first GLP-1 receptor agonist exenatide, and later lixisenatide, were based on the exendin-4 structure [25]. Exenatide and lixisenatide demand daily injections because of their renal excretion and short half-life [26]. GLP-1 mimetics are quickly degraded in the stomach’s acidic environment, and must be subcutaneously self-injected by the patient. Nowadays, GLP-1 receptor agonists are present in the form of modified human GLP-1, for instance, liraglutide, semaglutide, dulaglutide, and albiglutide; these are resistant to degradation by DPP-4, exhibit minimal renal excretion, and have to be subcutaneously self-injected by the patient only once a week (except liraglutide, which requires daily injections) [24]. Recently, an oral agent of GLP-1 receptor agonist semaglutide has been developed for the treatment of patients with T2DM [26]. Similar to native GLP-1, GLP-1 receptor agonists stimulate insulin secretion from beta cells, decrease glucagon release from alpha cells, and improve the insulin sensitivity of the peripheral tissue, resulting in better glucose homeostasis, evidenced by the reduction in hemoglobin A1c and fasting glucose level [27]. Therapy using GLP-1 receptor agonists also reduces weight via delaying gastric emptying and through the central nervous system promoting satiety [19,20]. Exendin-4 mimetics exenatide and lixisenatide are short-acting GLP-1 receptor agonists with high plasma concentrations at the time of meal intake, primarily targeting gastric emptying and postprandial hyperglycemia. On the contrary, the long-acting human GLP-1 receptor agonists liraglutide, albiglutide, dulaglutide, and semaglutide, and exendin-4 mimetic exenatide XR, have extended effects on GLP-1 receptors, with predominant impact on fasting glucose levels and hemoglobin A1. Long-acting GLP-1 receptor agonists have been found to reduce the effects on gastric emptying, resulting in less gastrointestinal side effects, such as diarrhea, nausea, and vomiting, but with stronger effects on weight reduction [28].

Liraglutide at a higher dose of 3 mg and semaglutide at a dose of 2.4 mg is used for the treatment of obesity in subjects without T2DM. Treatment with 3 mg liraglutide resulted in weight loss > 5% in 50–70% of the patients [29]. In June 2021, the United States Food and Drug Administration (FDA) put semaglutide on the market at the dose of 2.4 mg, for the treatment of obesity in adults. Semaglutide at a dose of 2.4 mg significantly reduced body weight by > 5% in 86.4% of patients, while almost 70% of treated patients showed weight loss > 10% [30]. Additional to their powerful effects on hyperglycemia and obesity, some GLP-1 receptor agonists provide cardiovascular protection and benefits. Following the publication of a meta-analysis showing that the antidiabetic drug rosiglitazone, beyond glucose control, increases the risk of myocardial infarction and cardiovascular death, since 2008, regulatory approval of therapies for T2DM also required cardiovascular safety [31]. The efficacy and safety of GLP-1 receptor agonists have been evaluated in eight clinical trials that included about 60,000 patients with T2DM [32]. To date, all cardiovascular outcome trials have shown noninferiority versus a placebo (both added to standard of care) against a primary endpoint of a three-point major adverse cardiovascular event (MACE), confirming the cardiovascular safety of these new drugs. The three-point MACE outcome was defined as the first occurrence of one of the following: death from a cardiovascular cause, nonfatal stroke, or nonfatal myocardial infarction. Nonfatal stroke and nonfatal myocardial infarction represented stroke and myocardial infarction that did not lead to death within 30 days after the occurrence. However, some cardiovascular outcome trials have shown superiority over a placebo against the same MACE endpoint, suggesting a cardioprotective action for these drugs [33]. The first reported trial, ELIXA, with exendin-4 mimetic lixisenatide conducted in patients with T2DM and recently diagnosed acute coronary syndrome was cardiovascular neutral for three-point MACE [34]. The second reported trial, LEADER, with modified human GLP-1 receptor agonist liraglutide, demonstrated a reduction in three-point MACE by 13%, driven mainly by a lower rate of death from cardiovascular disease in 9340 patients with T2DM and high cardiovascular risk [35]. The SUSTAIN 6 trial with injectable semaglutide demonstrated a reduction in three-point MACE driven mainly by a lower risk of nonfatal stroke in almost 40% in 3297 patients with T2DM and high cardiovascular risk [36]. The largest study was EXSCEL, with 2 mg long-acting extended-release exenatide, exendin-4 mimetic, which included 14,752 patients with T2DM. Compared to previous studies, this study included patients with and without a history of cardiovascular disease. Similar to exendin-4 mimetic lixisenatide, this study showed cardiovascular noninferiority [37]. The HARMONY trial with modified human GLP-1 receptor agonist albiglutide showed a reduction in three-point MACE driven mainly by a significant decrease in myocardial infarction by 25% in 9469 patients with T2DM and a history of cardiovascular disease [38]. The PIONEER 6 trial with oral semaglutide that included only 3183 patients comprising T2DM patients with established cardiovascular disease and patients with cardiovascular risk factors alone, showed cardiovascular noninferiority of oral semaglutide for three-point MACE [39]. The ongoing SOUL trial, which includes around 9640 patients with T2DM and established cardiovascular disease or risk factors, will confirm whether oral semaglutide has a favorable effect on three-point MACE, as seen with subcutaneous semaglutide [40]. Finally, the last published REWIND trial with dulaglutide conducted in 9901 patients with T2DM with either a previous cardiovascular event or cardiovascular risk factors showed a three-point MACE reduction by 12%, primarily achieved with nonfatal stroke reduction by 24% [41]. Taken together, treatment with GLP-1 receptor agonists are associated with a MACE reduction by 12%, cardiovascular death reduction by 12%, reduction in fatal or nonfatal stroke by 16%, reduction in fatal or nonfatal myocardial infarction by 9%, all-cause mortality was lowered by 12% as well as hospitalization for heart failure by 9% [42]. No significant side effects resembling pancreatic cancer, pancreatitis, or severe hypoglycemia were noted. Since GLP-1 receptor agonists have a similar chemical structure and a similar mechanism of action it is assumed that GLP-1 receptor agonists will have similar pharmacological and clinical effects (drug class effect). However, it seems that the GLP-1 receptor agonist has no unique class effect on cardiovascular outcomes, because human GLP-1 analogs (liraglutide, semaglutide, albiglutide, dulaglutide) have achieved superiority in cardiovascular safety studies compared to exendin-4 mimetics (lixisenatide, exenatide).

### 1.2. Glucose-Dependent Insulinotropic Polypeptide

GIP was the first incretin hormone; it was isolated in 1970–1975 and initially named gastric inhibitory peptide [15]. It was later renamed to glucose-dependent insulinotropic polypeptide because its insulinotropic effects were observed at physiological levels. Enteroendocrine K cells located in the small intestinal epithelium secrete GIP into circulation after oral food ingestion [43]. After secretion from the K cells, GIP binds to pancreatic beta cells and stimulates insulin secretion depending on the glucose level. GIP also stimulates the transcription of the beta cell insulin gene [44]. In patients with T2DM, the GIP effect on pancreatic beta cells and insulin secretion is reduced [45]. GIP receptors are also placed in pancreatic alpha cells. Compared to GLP-1, which suppresses glucagon secretion during hyperglycemia, GIP can stimulate glucagon secretion during low and high glycemia [46]. GLP-1 inhibits the release of glucagon via somatostatin release from pancreatic alpha cells, and GIP also stimulates somatostatin release. Although GIP stimulates glucagon secretion at elevated glucose levels, stimulatory effects on somatostatin release from pancreatic alpha cells might diminish this effect. Finally, the insulinotropic effect of GIP dominates during hyperglycemia, while the glucagonotropic effect of GIP seems to be most important during hypoglycemia [15]. Effects on the gastrointestinal tract are also opposite. While GLP-1 delays gastric emptying, GIP has no effects on stomach motility [47]. Similar to native GLP-1, GIP is quickly degraded by DPP-4, and has a half-life of only several minutes [48]. Similar to GLP-1 receptors, GIP receptors are also located in other tissues, such as the heart, adipose tissue, bone, adrenal cortex, cerebral cortex, olfactory bulb, pituitary, and hippocampus, although the GIP effects in these tissues are not fully understood [41]. GIP has anabolic effects on adipose tissue, reducing glucagon-mediated lipolysis and activating fatty acid synthesis, GIP inhibits osteoclastic bone resorption and induces the proliferation of osteoblasts, decreases gastrin-dependent acid secretion in the stomach, and has neuroprotective effects in Alzheimer’s disease [49,50]. GIP concentrations are increased in obese patients with T2DM because adipose tissue strongly augments GIP secretion; GIP increases the accumulation of body fat via lipoprotein lipase activity, increases conversion of fatty acids into triglycerides, and reduces lipolysis [51]. In the insulin resistance state characterized by high serum levels of glucose and insulin, GIP increases circulation in adipose tissue, resulting in higher triglyceride clearance [52]. In summary, GIP increases circulation in adipose tissue, increases triglyceride clearance from the circulation, and stimulates adipocyte lipid storage [22]. Mice without GIP receptors are lean without weight gain when fed a high-fat diet [53]. The fact that loss of GIP receptors may protect from diet-induced obesity has resulted in the evolution of GIP receptor antagonists for the treatment of obesity [54]. However, the beneficial effect of GIP receptor activation is difficult to understand, and the opposite effect was observed in preclinical animal studies, where GIP-receptor monoagonists lowered body weight. GIP-receptor monoagonists were found to be much less effective at reducing body weight in obese mice [55,56]. Contrary to GIP monotherapy, when administered with GLP-1 receptor agonists, its combination strongly decreases body weight [57]. In combination therapy, a portion of the weight effects of GIP can be attributed to enhanced GLP-1 receptor activity, and the beneficial effect of GIP may only be observed after metabolic control has been restored by GLP-1.

The effects of GIP on the cardiovascular system are not fully understood. However, compared to GLP-1 receptor agonists and their confirmed cardiovascular protection, and benefits in patients with T2DM, the possible benefits of GIP receptor agonism are questionable because of its impaired effects on insulin secretion and lower potency of weight loss [58]. These discouraging observations in animals have been partly reduced by the finding in diabetic and overweight individuals that GIP has central anorexic effects, which are possibly mediated by the GIP’s potential to improve the actions of GLP-1 [59,60]. GIP has opposite pro- and antiatherogenic effects on different types of endothelial cells. GIP receptors are expressed in monocytes that differentiate into macrophages in vessel walls, and are components of atherosclerosis plaque [61]. GIP infusion reduces macrophage infiltration into plaque, its accumulation, and plaque formation, and increases the collagen content of aortic plaques, which consequently stabilizes atherosclerotic plaque [62]. The infusion of active GIP also suppresses neointimal hyperplasia caused by injury and proliferation of the vascular cell, which may inhibit restenosis followed by angioplasty [63]. However, in animal models of inactivated GIP receptors, interstitial fibrosis and left ventricular cardiomegaly was diminished and harmful effects in myocardial infarction were reported [64]. Anti-inflammatory effects after activation of GIP receptors include decreased adipose tissue inflammation and blood interleukin-6 (IL-6) levels, and increased serum adiponectin level and adipose tissue expression, while inhibition of GIP receptors results in higher blood and adipose tissue levels of IL-6 and higher gingival inflammation [65]. Daily GIP injections decrease several proinflammatory cytokines, such as interleukin-1 beta, IL-6, and tumor necrosis factor (TNF)-alpha in mice, while the level of anti-inflammatory and insulin-sensitizing adipokine adiponectin is enhanced [66]. While GIP demonstrates several antiatherogenic actions, increases nitric oxide production, increases insulin sensitivity of white adipose tissue, inhibits proliferation of vascular smooth muscle cells, and suppresses inflammatory responses of adipocytes, monocytes, and macrophages, it also increases adipocyte inflammation [67].

### 1.3. Dual GIP and GLP-1 Receptor Agonists

As mentioned above, the first isolated incretin hormone was GIP; however, it was not used as a therapy for T2DM because its function was not fully understood in the state of hyperglycemia. Recently, the synergistic GLP-1 and GIP action have been found to be beneficial in glucose control and weight loss [49]. Dual GIP and GLP-1 receptor co-activation enhances insulin secretion by 20–30% more than the single administration, improves insulin sensitivity, and reduces glucagon secretion, and the reduction in hyperglycemia additionally reduces the resistance to GIP [68]. In a hyperglycemic state, insulin secretion increases with dual GIP and GLP-1 co-infusion, simultaneously inhibiting glucagon secretion to a much higher extent compared to separate infusion or glucose infusion alone [69]. In addition to the common synergistic effects on insulin secretion and synthesis, dual GIP and GLP-1 therapy more strongly stimulate genes involved in beta cell differentiation and survival compared to individual components [70]. While GLP-1 infusion suppresses glucagon secretion, and GIP promotes glucagon response, dual combined infusion did not change glucagon level [71]. In animal studies, dual GIP and GLP-1 stimulation also have additional synergistic effects in reducing weight and fat mass, and food intake [72].

The beneficial effect of dual GIP and GLP-1 receptor agonists have been confirmed in human trials. NNC0090-2746 is a dual GIP and GLP-1 receptor agonist developed by Novo Nordisk. A clinical study that included patients with T2DM treated with metformin, compared to patients treated with liraglutide, showed a comparable reduction in hemoglobin A1c, but a significantly higher reduction in body weight [68]. In 2020, Novo Nordisk stopped further research on dual GIP and GLP-1 receptor agonists NNC0090-2746 because single GLP-1 receptor agonist semaglutide in a dose of 2.4 mg showed superior weight loss effects [73]. LY3298176 is a dual GIP and GLP-1 receptor agonist developed by Eli Lilly and Company. Compared to GLP-1 receptor agonist dulaglutide and a placebo, LY3298176 significantly reduced hemoglobin A1c and body weight with similar adverse effects to dulaglutide [74]. LY3298176 is administered once weekly with subcutaneous injection, and has fivefold higher potency at human GIP receptors [75]. After encouraging first clinical results from LY3298176, a phase 3 clinical trial program with the name tirzepatide, an agent that activates both the GIP receptor and GLP-1 receptor, was started [75]. Tirzepatide was subcutaneously injected in a dose of 5, 10, or 15 mg, and after up to 1 year of treatment 81–97% of patients with T2DM achieved a hemoglobin A1c < 7% and 23–62% of patients achieved hemoglobin A1c < 5.7%. After 40 weeks of treatment with tirzepatide 15 mg, 47–57% of patients with T2DM lost over 10% of body weight compared to the placebo group [22].

SURPASS 1 investigated monotherapy of tirzepatide in patients with T2DM and increased hemoglobin A1c on diet and exercise and found a superior dose-dependent reduction in hemoglobin A1c and body weight [76]. SURPASS-2 compared treatment with tirzepatide and weekly GLP-1 receptor agonist semaglutide in patients with T2DM, and found that tirzepatide was noninferior and superior to semaglutide in reduction in the hemoglobin A1c in all doses, but with much higher weight loss [9]. The results of SURPASS-3 and -4 studies showed a higher reduction in hemoglobin A1c and body weight with tirzepatide compared to basal insulin degludec in patients with T2DM with or without cardiovascular disease [77,78]. The SURPASS-5 treatment with tirzepatide resulted in a significantly higher reduction in hemoglobin A1c and body weight compared to the placebo when added to basal insulin glargine [79]. Finally, SURPASS AP-Combo, which includes patients taking metformin with or without a sulfonylurea, and compares tirzepatide versus insulin glargine, and SURPASS-CVOT, a cardiovascular trial that compares cardiovascular safety of tirzepatide against 1.5 mg dulaglutide, are ongoing [49]. In addition to glucose control and weight loss, the majority of patients with T2DM have atherogenic dyslipidemia, and thus typically present with low levels of high-density lipoprotein (HDL) cholesterol and high triglycerides. There are also structural changes in low-density lipoprotein (LDL) cholesterol resulting in the predominance of atherogenic small dense LDL cholesterol, which is secondary to metabolic syndrome and insulin resistance [80]. In a study that investigated the effects of tirzepatide therapy on serum lipids, the tirzepatide reduced the serum large triglyceride-rich lipoprotein particles, atherogenic apoB, apoC-III, and small dense LDL-cholesterol levels compared with dulaglutide therapy and placebo [9]. Higher doses of tirzepatide also significantly ameliorate biomarkers of nonalcoholic steatohepatitis while increasing adiponectin levels in patients with T2DM [81]. The direct and indirect effects of GIP and GLP-1 are presented in Table 1. These impressive results have generated great interest in dual GIP and GLP-1 receptor co-agonists, although the relative direct and indirect contributions of GIP receptor agonists need to be better understood [82]. In addition, it has to be determined how semaglutide, at a higher dose than 2.4 mg, which has recently been approved for the treatment of obesity, compares with tirzepatide [83].

### 1.4. Nephroprotective Properties of the GIP and GLP-1

The dose of the older incretin-mimetics, such as exenatide and lixisenatide, must be reduced in patients with renal insufficiency because they are eliminated mainly by glomerular filtration. Exendin-4 analogs are contraindicated in patients with chronic kidney disease (glomerular filtration rate (GFR) below 30 mL/min/1.73 m^2^) because of the risk of accumulation and toxicity. On the contrary, newer human-like GLP-1 receptor agonists are metabolized locally in the target tissues because they possess a large molecule that is protected from renal clearance and has no principal place of elimination [84]. Semaglutide has similar application in patients with normal, mild, moderate, and severe renal disease [85]. Thus, human GLP-1 analogs can be used in a maximum tolerated dose in subjects with GFR up to 15 mL/min/1.73 m^2^ [40]. The human GLP-1 receptor agonists semaglutide, liraglutide, and dulaglutide successfully improved glycemic regulation in subjects with T2DM, and significantly reduced renal function, even in those on dialysis [86,87,88]. In addition, in patients with T2DM and moderate to severe reduced estimated GFR, GLP-1 receptor agonist dulaglutide reduced hemoglobin A1c similarly to basal insulin glargine, but with the added benefit of weight loss, lower rate of hypoglycemia, and a reduced decline in renal function [89]. Similar results were obtained in patients with T2DM and moderate renal disease when comparing oral semaglutide to a placebo [90]. Since dual GIP/GLP-1 receptor agonists have only recently been used for the treatment of T2DM, there is limited knowledge about their renal effects. GIP concentrations are higher in uremic patients, and in patients with severe renal disease, GIP has a 10% to 30% lower capacity to increase insulin release [90,91]. The molecular weight of tirzepatide is 4.8 kDa, which is significantly below the glomerular filtration barrier of 30–50 kDa. A recently published study investigating tolerability and pharmacokinetics of tirzepatide found that the pharmacokinetics of tirzepatide were similar between subjects with different degrees of renal failure compared with healthy subjects, and with mild gastrointestinal adverse events in subjects with renal impairment [92]. The authors suggested that the adjustment of the dose of tirzepatide is not needed in patients with renal disease and even in the dose for dialysis.

**Figure 2:**
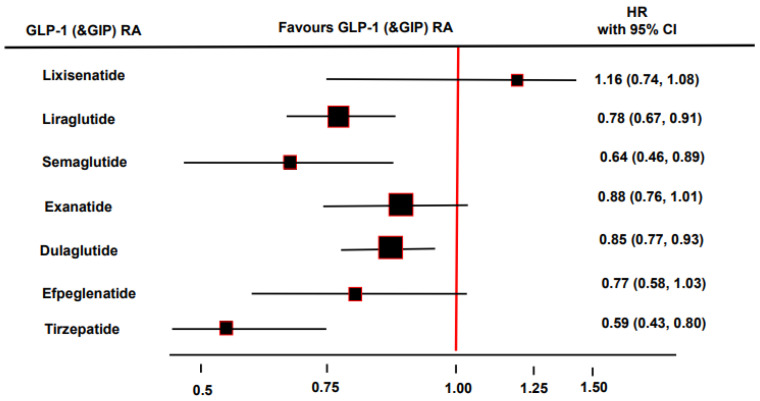
Renal endpoints of single glucagon-like peptide 1 (GLP-1) and dual GLP-1 and glucose-dependent insulinotropic polypeptide (GIP) receptor agonists (RA) [35,36,41,91,98,99,100].

**Figure 3:**
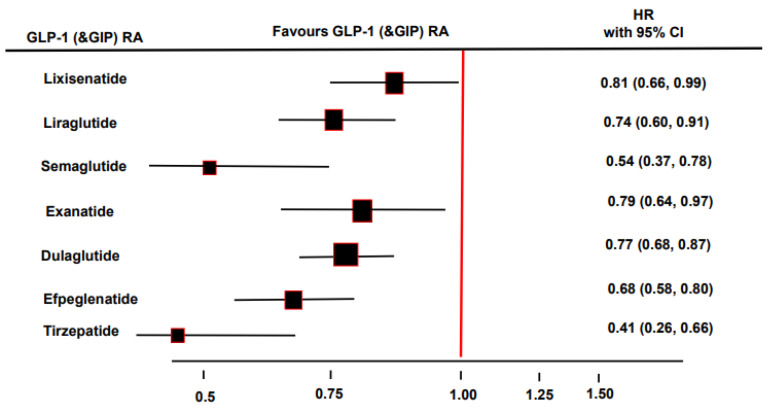
Single glucagon-like peptide 1 (GLP-1) and dual GLP-1 and glucose-dependent insulinotropic polypeptide (GIP) receptor agonists (RA) effects on macroalbuminuria [35,36,41,91,98,99,100].

GLP-1 receptor agonist-based cardiovascular outcome trials also had secondary kidney disease outcomes. The LEADER trial, with modified human GLP-1 receptor agonist liraglutide, included 23% of patients with a history of chronic kidney disease, and demonstrated a reduction in kidney failure, doubling of the serum creatinine, death from kidney disease, and a lowered risk of macroalbuminuria by 26% [35]. Liraglutide therapy has no beneficial effects on diabetic retinopathy, another microvascular complication of diabetes. SUSTAIN-6 was a cardiovascular outcome trial that included semaglutide, and revealed beneficial effects on renal function, with a 46% lower risk of development of macroalbuminuria [36]. Surprisingly, a group of patients treated with semaglutide exhibited a significantly higher rate of diabetic retinopathy complications compared with the placebo group. REWIND was a cardiovascular outcome trial that included dulaglutide in which a significant 15% risk reduction in the renal composite endpoint compared to placebo was observed, similar to the effects of liraglutide and semaglutide, driven mainly by a 23% lower risk of development of macroalbuminuria [41]. In the AMPLITUDE-O trial, weekly subcutaneous injections of GLP-1 receptor agonist efpeglenatide (4 or 6 mg) for a median of 1.8 years led to a 27% lower risk of incident MACE and a 32% lower risk of a composite renal outcome event compared to a placebo in patients with T2DM and either a history of cardiovascular disease or current kidney disease [93] (Figure 2). In the AWARD-7 trial, which included patients with T2DM and chronic kidney disease, treatment with dulaglutide compared to insulin resulted in a lower rate of GFR reduction or end-stage renal disease, particularly in those with macroalbuminuria [94]. In the ELIXA trial, exendin-4 analog lixisenatide, compared to a placebo, reduced the progression of albuminuria in patients with macroalbuminuria [95]. In the EXSCEL trial, exendin-4 analog exenatide, compared to placebo, decreased the risk of the onset of macroalbuminuria, dialysis or transplantation, renal death, and risk of GFR decline by 40% [96]. Finally, dual GLP-1 and GIP receptor agonist tirzepatide reduced the risk of a composite kidney outcome (estimated GFR decline of 40% or more, renal death, progression to end-stage renal disease, or new-onset macroalbuminuria) by 41% compared to insulin glargine (HR 0.59, 95% CI 0.43–0.80). Renal protection was predominantly achieved with a reduction in the risk of new-onset macroalbuminuria by 59% (HR 0.41, 95% CI 0.26–0.66) [97] (Figure 3).

Activation of a proinflammatory state, increased production of advanced glycation end products (AGEs), and enhancement of oxidative stress are all disturbances connected with hyperglycemia and metabolic changes typical of diabetes. However, inflammation is also an underlying condition in the development of diabetic nephropathy [12]. Apoptosis of podocytes in the kidney related to proteinuria and glomerulosclerosis is induced by higher levels of proinflammatory advanced oxidation protein products (AOPPs), which are increased in patients with diabetes [98]. AOPPs are related to diabetic nephropathy by promoting the production of several other proinflammatory factors, such as nicotinamide adenine dinucleotide phosphate (NADPH) oxidase, nuclear factor kappa-light-chain-enhancer of activated B cells (NF-κB), and reactive oxygen species. Characteristics of anatomic manifestations of diabetic nephropathy include mesangium growth and disseminate thickening of the basement membranes, endothelial edema, loss of podocytes, tubulointerstitial fibrosis, and accumulation of inflammatory cells, as well as the deposition of proteins and hyalin in the subendothelial and arterial area [99]. In addition to those previously mentioned, the majority of patients with T2DM have many others risk factors for chronic kidney disease, such as insulin resistance and obesity, high blood pressure, and dyslipidemia [100].

In addition to the pancreas, GIP receptors are present in many other tissues, including the heart, bone tissue, adipose tissue, adrenal cortex, cerebral cortex, pituitary, and hippocampus, but not in kidneys; this excludes the potential for direct effects on kidney function [15]. However, GIP effects on inflammatory processes have been widely investigated [65]. Anti-inflammatory effects after activation of GIP receptors include decreased adipose tissue inflammation and serum IL-6 levels, and increased serum adiponectin levels and adipose tissue expression, while inhibition of GIP receptors increases IL-6 serum and adipose tissue levels [65]. Adiponectin has anti-inflammatory effects and restores insulin sensitivity in diabetes and other metabolic disturbances connected with obesity. In addition, adiponectin receptor agonist AdipoRon showed beneficial effects on diabetic nephropathy [101]. On the contrary, higher levels of serum IL-6, an important inflammatory mediator, are associated with worsening of kidney function in diabetes [102]. Daily GIP injections decrease several proinflammatory cytokines, such as IL-6, interleukin-1 beta, and TNF-alpha, in animal models, while adiponectin level increases [66]. Proinflammatory interleukin-1 beta levels and interleukin-1 beta gene polymorphism is also associated with the risk of development and progression of kidney disease in T2DM [103,104]. TNF-alpha is a cytokine that induces inflammatory processes and cell death, modulates immune responses; furthermore, TNF-alpha blockade ameliorates diabetic nephropathy in rats [105]. Serum TNF-alpha levels are positively associated with albuminuria and negatively associated with GFR, and are independently associated with all-cause mortality and declining GFR up to end-stage renal disease [106]. While GIP has several antiatherogenic actions, increases nitric oxide production and insulin sensitivity of white adipose tissue, inhibits proliferation of vascular smooth muscle cells, and suppresses inflammatory responses of adipocytes, monocytes, and macrophages, it also increases adipocyte inflammation [67]. GIP has many other indirect protective effects on kidney function. Stimulating cerebral receptors reduce food intake and body weight. Acting on subcutaneous white adipose tissue, GIP increases insulin sensitivity and blood flow, increases lipid buffering capacity and storage capacity, and decreases proinflammatory immune cell infiltration. Acting on skeletal muscle, GIP increases insulin sensitivity and decreases ectopic lipid accumulation. Finally, systemic effects include a decrease in hyperglycemia and dyslipidemia [65].

Compared to GIP, GLP-1 receptors present in the proximal tubular cells and preglomerular vascular smooth muscle cells in the kidney have direct effects on renal function [107]. Activation of GLP-1 receptors on glomerular capillary and vascular walls upregulates the production of a major second messenger cyclic adenosine monophosphate (cAMP), which then triggers protein kinase A (PKA) activity. The upregulated cAMP and PKA inhibit oxidative renal injury, one of the leading causes of diabetic nephropathy, via the inhibition of NADPH oxidase, a major source of superoxide anion activated under chronic hyperglycemia [108]. The exclusive expression of GLP-1 receptors in the vascular wall of arterioles and arteries in the kidney induces the dilation of vessels supplying the glomeruli [109]. GLP-1 receptors are also expressed in renin-secreting cells of the juxtaglomerular apparatus. Accordingly, the direct renal benefit of GLP-1 receptor agonists includes increased natriuresis and reduced hyperfiltration, improved tubule-glomerular feedback, decreased renal and systemic inflammation, decreased angiotensin II and renin concentration, decreased glomerular atherosclerosis and renal hypoxia, and endothelial-dependent vasodilation [110,111]. GLP-1 inhibits sodium–hydrogen exchanger 3 (NHE3) localized at renal proximal tubular cells, inducing natriuresis and diuresis, with a mild influence on tubuloglomerular feedback [112]. GLP-1 receptor agonists decrease serum angiotensin II involved in renal sodium wasting and, consequently, decrease blood pressure [113]. Animal studies suggest direct effects on kidney function via atrial natriuretic peptide [110]. GLP-1 receptor agonists may decrease glomerular hyperfiltration by inhibiting angiotensin II and endothelin-1, causing vasoconstriction, although this was not confirmed in healthy overweight men and patients with T2DM [36,114,115,116]. Treatment with lixisenatide, compared to basal insulin glargine, does not affect postprandial renal hemodynamics in patients with T2DM without renal disease [117]. GLP-1 receptor agonists also downregulate the expression of several proinflammatory biomarkers in animal models with diabetic nephropathy, such as TNF-alpha, collagen I, fibronectin, monocyte chemoattractant protein-1(MCP-1), and alpha-smooth muscle actin (α-SMA), and reduce the proinflammatory reaction by inhibiting accumulation of inflammatory cell, blocking profibrotic signaling and the activation of the mononuclear phagocyte system [1,32,118]. Exendin-4 and liraglutide suppressed oxidative stress and proinflammatory cytokine production and reduced the expression of transforming growth factor-beta 1 (TGF-beta 1), NF-κB, intercellular adhesion molecule 1 (ICAM1), and reduced macrophage accumulation in the kidney [119,120]. Additional to GLP-1 receptor expression in pancreatic cells, neurons, and in the cardiovascular system, the expression of GLP-1 receptors in immune cells has not been previously determined. Recently, in animal models, the expression of GLP-1 receptors in different subpopulations of macrophages has been described [121]. GLP-1 receptor deficiency increases IL-6 production and macrophage polarization, resulting in the migratory ability of the macrophages.

**Figure 4:**
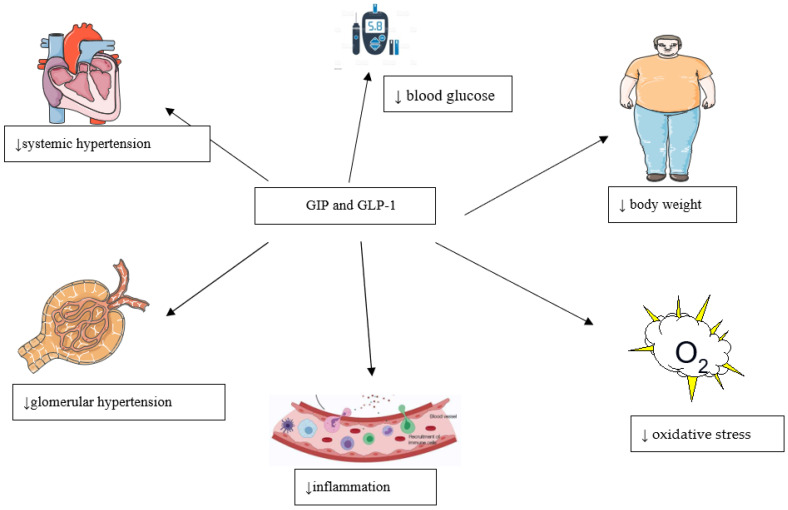
Potential nephroprotective effects of glucose-dependent insulinotropic polypeptide (GIP) and glucagon-like peptide-1 (GLP-1) receptor agonists.

Additional to improving conventional risk factors of chronic kidney disease, such as hyperglycemia, hypertension, and weight, GLP-1 receptor agonists also have indirect nephroprotective effects. Moreover, as well as improving blood glucose, serum lipids, weight, and blood pressure, GLP-1 receptor agonists possess anti-ischemic and anti-inflammatory properties (Figure 4) [110]. In the LEADER study with liraglutide, the positive effects on renal function existed even after adjustment for risk factors such as hypertension, hyperglycemia, and body weight [35]. Additional to the well-known effects on glycemia and weight loss, treatment with GLP-1 receptor agonists reduce systolic blood pressure by 2.22 mmHg, independently of weight loss or improvement in hemoglobin A1c, according to results of a meta-analysis that included 26,654 patients from 33 clinical trials [122]. It is well known that hypertension increases the risk of diabetic nephropathy onset and progression and cardiovascular morbidity and mortality [123]. In addition, systolic hypertension in patients with chronic kidney disease, compared to diastolic hypertension, imposed a high risk of tissue damage, independent of age [124]. Treatment with GLP-1 receptor agonists also mildly reduces total and LDL cholesterol and triglycerides [125]. Diabetic dyslipidemia is not only a consequence of kidney dysfunction, but the underlying condition in the pathogenesis and progression of diabetic nephropathy; furthermore, levels of serum triglyceride, and total and LDL cholesterol, are high in subjects with diabetic nephropathy compared to control subjects [126,127].

## 2. Conclusions

Diabetes mellitus with hypertension is the leading cause of diabetic nephropathy. Incretin hormones, a class of antidiabetic drugs, have several beneficial effects on the human body, in addition to their effects on blood glucose. The main incretins are GLP-1 and GIP. GLP-1 receptor agonists are a well-established antidiabetic drug that, along with glucose and weight reduction, also improve cardiovascular and renal outcomes in patients with T2DM; these outcomes are not completely associated with reducing hyperglycemia. Recently, the synergistic action of GLP-1 and GIP has attracted scientific interest. GLP-1 activity is systemic, while GIP activity is mainly pancreatic. Co-administration of GLP-1 and GIP additionally reduces blood glucose and body weight compared with monotherapy with each hormone. While GLP-1 receptor agonist has been proven to provide cardiorenovascular protection, studies conducted on animals and in vitro suggest that there are possible renoprotective actions of GIP in humans, because GIP influences several mechanisms that take part in diabetic nephropathy development and progression by reducing proinflammatory and profibrotic states factors. Recently, dual GIP and GLP-1 receptor agonist semaglutide have been approved for the treatment of T2DM. Compared to single GLP-1 receptor agonist semaglutide, dual GIP and GLP-1 receptor agonist tirzepatide was associated with a superior reduction in hemoglobin A1c and body weight, and improved kidney outcomes in adults with T2DM with increased cardiovascular risk. Further investigation must be conducted to elucidate the exact nephroprotective mechanism and properties of the dual GIP and GLP-1 receptor agonists.

## Figures and Tables

**Table 1:** Direct and indirect effects of the glucose-dependent insulinotropic polypeptide (GIP) and glucagon-like peptide-1 (GLP-1).

Direct Effects	Indirect Effects
↑ satiety (GLP-1) ↓ food intake (GLP-1 and GIP) ↓↑ nausea (↑ GLP-1, ↓ GIP) ↑ insulin secretion (GLP-1 and GIP) ↑↓ glucagon secretion(↑ GIP, ↓GLP-1) ↓ gastric emptying (GLP-1) ↑ insulin sensitivity in adipose tissue (GIP) ↑ lipid buffering capacity (GIP) ↑ blood flow in adipose tissue (GIP) ↑ storage capacity of adipose tissue (GIP) ↓ proinflammatory immune cell infiltration in adipose tissue (GIP) ↑ natriuresis (GLP-1) ↓ hyperfiltration (GLP-1) ↑ tubule-glomerular feedback (GLP-1) ↓ renal and systemic inflammation (GLP-1) ↓ renin and angiotensin 2 (GLP-1) ↓ oxidative stress (GLP-1) ↓ renal hypoxia (GLP-1)	↓ hyperglycemia (GLP-1 and GIP) ↓ body weight (GLP-1 and GIP) ↓ dietary triglycerides (GIP) ↑ insulin sensitivity in the liver (GLP-1) ↑ insulin sensitivity in muscle (GLP-1) ↓ hepatic glucose production (GLP-1) ↓ ectopic lipid accumulation in the liver (GLP-1) ↓ ectopic lipid accumulation in muscle (GIP) ↑ metabolic flexibility in muscle (GIP) ↓ blood pressure (GLP-1) ↓ neointimal formation (GLP-1) ↑ coronary flow (GLP-1) ↓ intestinal lipid uptake (GLP-1) ↓ bone resorption (GIP)

## Data Availability

Not applicable.
